# Potential Use of Plant Waste from the Moth Orchid (*Phalaenopsis* Sogo Yukidian “V3”) as an Antioxidant Source

**DOI:** 10.3390/foods6100085

**Published:** 2017-09-27

**Authors:** Truong Ngoc Minh, Phung Thi Tuyen, Do Tan Khang, Nguyen Van Quan, Pham Thi Thu Ha, Nguyen Thanh Quan, Yusuf Andriana, Xinyan Fan, Truong Mai Van, Tran Dang Khanh, Tran Dang Xuan

**Affiliations:** 1 Graduate School for International Development and Cooperation, Hiroshima University, Hiroshima 739-8529, Japan; minhtn689@gmail.com (T.N.M.); phungtuyen@gmail.com (P.T.T.); dtkhang@ctu.edu.vn (D.T.K.); nguyenquan26@gmail.com (N.V.Q.); phamthithuhabt@gmail.com (P.T.T.H.); quanagi@gmail.com (N.T.Q.); yusufandriana@yahoo.com (Y.A.); xinyanfan5160@gmail.com (X.F.); truongmaivan1991@gmail.com (T.M.V.); 2 Division of Genetic Engineering, Agricultural Genetics Institute, Pham Van Dong Street, Hanoi 10000, Vietnam; khanhkonkuk@gmail.com

**Keywords:** *Phalaenopsis* Sogo Yukidian “V3”, antioxidant activity, moth orchids, phenolic acids, plant part waste

## Abstract

This research was conducted to exploit the waste of used plant parts from the widely marketed moth orchid cultivar (*Phalaenopsis* Sogo Yukidian “V3”). Various extracts of roots, stems, and leaves were evaluated for total phenolics, total flavonoids, and antioxidant capacity. The bound extract from stems contained the highest total phenolics (5.092 ± 0.739 mg GAE (gallic acid equivalent)/g DW (dry weight)). The maximum total flavonoids (2.218 ± 0.021 mg RE (rutin equivalent)/g DW) were found in the hexane extract of leaves. Ethyl acetate extract from roots showed the greatest antioxidant activity compared to other extracts. Of these extracts, the IC_50_ values of these samples were 0.070 mg/mL, and 0.450 mg/mL in a free radical 1,-diphenyl-picryl-hydrazyl (DPPH) assay and reducing power method, respectively. The lipid peroxidation inhibition (LPI) was found to be 94.2% using the *β*-carotene bleaching method. Five phenolic compounds including caffeic acid, syringic acid, vanillin, ellagic acid, and cinnamic acid were quantified by high performance liquid chromatography (HPLC). It is suggested that the roots of the hybrid *Phalaenopsis* Sogo Yukidian “V3” cultivar may be exploited as an effective source of antioxidants.

## 1. Introduction

Phytoplankton is a rich source of phytochemicals and antioxidants. A considerable number of studies have been conducted regarding new forms of antioxidants due to their known health benefits [1]. Natural antioxidants are an important source for preventing or scavenging free radicals and reducing chronic and degenerative diseases by slowing the aging process and treating cancer. Secondary metabolites, of which phenolic compounds are a key component, have diverse applications in antioxidant production. Thus, recent replacement of synthetic antioxidants with natural compounds and the identification of new antioxidants have become very important [2].

The genus *Phalaenopsis* (moth orchids) comprises about 66 species belonging to the *Orchidaceae* family (estimated > 25,000 species) [3,4]. Among them, the *Phalaenopsis (Phal.)* Sogo Yukidian “V3” cultivar (moth orchid), which is a hybrid of *Phal.* Yukimai crossed with *Phal*. Taisuco Kochdian varieties, is white-colored, has a large flower, and is common in the market [4]. The *Phal.* crinkled flowers indicates relatively short flower longevity, with young seedlings typically propagated by tissue culture [5]. This cultivar of orchid is also a popular potted plant in Japan, America, and Europe [6]. The plant becomes very weak after flowering one or two times, subsequently turning into waste [2]. Moreover, although this orchid has great economic value, its disposal has proven to bring about detrimental effects on the environment [2,7,8]. Therefore, the objective of this study was to evaluate the antioxidant properties of plant part waste of the moth orchid.

Phenolic compounds exist in both free and bound forms in plant cells. Free phenolic compounds are extractable with solvents, while bound phenolic compounds are covalently bound to the plant matrix [9]. Therefore, different extraction techniques using increasingly polar solvents, including hexane, chloroform, ethyl acetate, and water, were applied to examine their efficacy for extracting antioxidants and evaluate the antioxidant capacity, total phenolics, and total flavonoids in the orchid. The identification and quantification of individual phenolic acids were also conducted.

## 2. Materials and Methods

### 2.1. Materials

The hybrid *Phal.* Sogo Yukidian “V3” cultivar used in this study was grown in a greenhouse at the Kurousu Orchid Company, Saitama Prefecture, Japan from April 2014 to July 2015. After flowering, the whole plant was collected and used for the experiment. Leaves, roots, and stems of this hybrid orchid were cut separately into small pieces and dried in an oven at 30 °C. After drying, the samples were ground into powder using a kitchen grinder.

### 2.2. Preparation of Free Phenolics and Bound Phenolics

Different extracting solvents with increasing polarity were used to examine the efficacy of different extracts on orchid plant parts. The solvents used included the following compounds: hexane, chloroform, ethyl acetate, and water (Table 1). Dried plant part powder was first separated into groups of stems, roots, and leaves. Next, these groups were placed in ethanol and were concentrated under reduced pressure at 40 °C. The resulting dried ethanol extracts were then dissolved in water and successively extracted with each of the solvent compounds, hexane, chloroform, and ethyl acetate. The resulting mixtures were added to the aqueous solution that remained following extraction. These mixtures were filtered and concentrated under decreased pressure to obtain extracts in hexane, chloroform, ethyl acetate, and aqueous extracts [10]. The ethanol residues were hydrolyzed with 4 M NaOH at 50 °C and extracted with ethyl acetate to obtain bound phenolics [11].

### 2.3. Total Phenolic Content

The Folin—Ciocalteu method was used to determine the phenolic content of both free phenolics or bound phenolics of plant extracts, of which, the total phenolic content was calculated using a calibration curve and presented as mg of gallic acid equivalent (GAE)/g dry weight (DW) [12].

### 2.4. Estimation of Flavonoid Content

The aluminum chloride colorimetric assay was used to determine the flavonoid content of both free phenolics and bound phenolics of plant extracts, of which, the total flavonoid content was established using a calibration curve and expressed as mg rutin equivalent/g DW [13].

### 2.5. Antioxidant Activity Measurement by DPPH Scavenging Assay

Experiments were carried out according to the method described in Elzaawely et al. [14]. The reaction mixture of 0.5 mL of sample extract, BHT (butylated hydroxytoluene) (positive control), or MeOH (methanol) (control), 0.25 mL of 0.5 mM DPPH, and 0.5 mL of 0.1 M acetate buffer (pH 5.5), were kept in the dark at 26 °C for 30 min. A HACH DR/4000U spectrophotometer (Hach, Loveland, CO, USA) was used to evaluate the absorbance at 517 nm. The rate of inhibition percentage was determined according to the formula: % radical scavenging activity = [(A_control_ − A_test_)/A_control_] × 100. The inhibitory concentration (IC_50_) of the sample extracts is the amount needed to inhibit 50% of the DPPH radicals. As such, lower IC_50_ values show higher antioxidant activity.

### 2.6. Antioxidant Activity Measurement by β-Carotene Bleaching Method

Experiments were carried out according to the method described by Siddhuraju and Becker [15]. A one mL solution of *β*-carotene (2.0 mg) in 10 mL chloroform, 20 μL linoleic acid, and 200 mg Tween-40 was mixed. An aliquot of 50 mL of oxygenated water was added after concentration under reduced pressure at 45 °C. After shaking, 1 mL the *β*-carotene linoleic acid emulsion was mixed with 120 mL (1000 ppm) of sample extracts, BHT (positive control), or MeOH (control). The samples were then incubated at 50 °C. The absorbance of each mixture was measured by spectrophotometer at 492 nm, at an interval of 15–180 min to quantify the absorbance for all samples. Lipid peroxidation inhibition (LPI) percentage was calculated using the following formula [16]: % LPI = A_1_/A_0_ × 100.

A_0_ corresponds to the absorbance value estimated at time zero for the test sample, and A_1_ corresponds to the absorbance value at 180 min.

### 2.7. Reducing Power

The reducing power was determined following previously described methods [17]. A reaction mixture of 1 mL of either extract or BHT (with concentrations 25, 50, 100, and 250 ppm in MeOH) and 2.5 mL phosphate buffer (0.2 M, pH 6.6) and 2.5 mL potassium ferricyanide [K_3_Fe(CN)_6_] (10 g/L) was prepared. Next, the solution was incubated at 50 °C for 30 min. Following incubation, a total of 2.5 mL trichloroacetic acid (100 g/L) was added to the mixture, which was then centrifuged at 4000 rpm for 10 min. Finally, the supernatant solution (0.5 mL), distilled water (0.5 mL), and 0.5 mL FeCl_3_ (1 g/L) was measured for absorbance at 700 nm. The absorbance of the reaction mixture is directly proportional to reducing power.

### 2.8. HPLC Analysis

Phenolic compound analyses were carried out using HPLC by a method described in Xuan et al. [18]. Five mL of each sample extract was first filtered using a 0.2 μm filter (KANTO Chemical, Tokyo, Japan) and then injected into the HPLC (JASCO PU-2089 Plus, JASCO Corporation, Tokyo, Japan, column J–Pak Symphonia C18 column; 225 mm × 4.6 mm., i.d., 5 μm, 100 Å (Å is an unit of length equal to 1 × 10-10 meters (m) or 0.1 nanometer (nm)). A gradient elution of two solvents including absolute methanol (A) and 0.1% acetic acid (B) was used. The initial step began with the mobile phase A with gradient concentration from 5% to 10% in the first 5 min, then increased from 10% to 90% for next 45 min, and 100% for the last 10 min; wavelength: 254 nm; flow rate: 1.0 mL/min. Each sample was measured in triplicate, and each phenolic compound was identified and quantified in comparison with retention times and peak areas of their respective standards.

### 2.9. Statistical Analysis

All data from this research were analyzed using Minitab Software (version 16.0, copyright 2015, Minitab Inc., State College, PA, USA) which performed a two-way analysis of variance (ANOVA) with *p* < 0.05. All trials were conducted using a completely randomized block design with at least three replicates.

## 3. Results

### 3.1. Total Phenolic Content (TPC) and Total Flavonoid Content (TFC)

**Figure 1:**
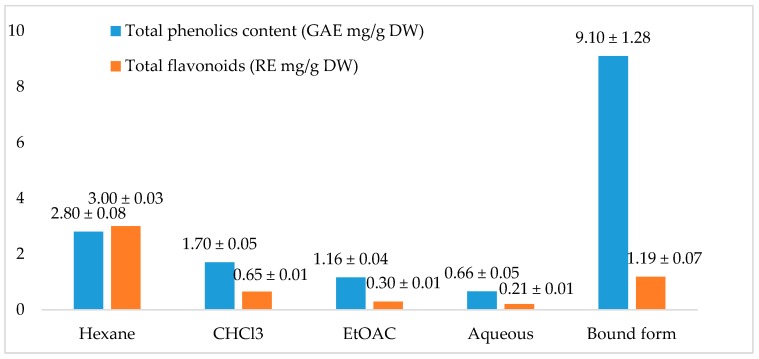
Total phenolic and flavonoid content in extracts of plant parts of *Phal.* Sogo Yukidian “V3” cultivar. Values represent each mean ± standard deviation (SD) (*p* < 0.05) (*n* = 3); GAE: gallic acid equivalent; RE: rutin equivalent; DW: dry weight.

Different chemicals have various protective and therapeutic effects and may help in the prevention of illness and in maintaining good health [19]. Among them, flavonoids and phenolics are highly effective antioxidants. Some diseases have been treated through the application of polyphenols and flavonoids associated with free radical based terminators, and they have been reported to be effective in disease control as well as involvement in many physiological functions [20,21]. Phenolic compounds have been highlighted as antioxidants, which act as free radical oxidant binders, having been shown to elicit pharmacological activity and express biological properties [22,23]. Das et al. [24] mentioned that some flavonoids containing hydroxyls contribute to the narrowing effect of the roots of many plants. The flavonoid mechanism of action is accomplished through either scavenging or chelating. It is well known that many plants are highly effective in the collection of free radicals and antioxidants. Thus, plant phenolics are generally highly effective in removing free radicals [25]. The contents of total phenolics and flavonoids in hexane, chloroform, ethyl acetate, and aqueous extracts prepared from the whole plant of the *Phal.* Sogo Yukidian “V3” cultivar vary between 0.66 and 9.10 mg GAE/g DW and 0.21 to 3.00 mg rutin equivalent (RE)/g DW, as shown in Figure 1. These results suggest that the highest amount of phenolics was detected in bound extract in comparison with flavonoids and were observed principally in the hexane extracts.

### 3.2. Antioxidant Activity by the DPPH Radical Scavenging Assay

At room temperature, a stable, free radical DPPH creates a violet solution in ethanol, while an antioxidant shows a colorless solution in aqueous ethanol [2]. DPPH is an easy and accurate way to determine antioxidant presence in plant specimens [14]. DPPH reagents provide a convenient and accurate method for titrating a collective oxidation of natural or synthetic antioxidants [26]. The DPPH free radical scavenging activity of the orchid extracts is shown in Table 2, and is exhibited through the IC_50_ value, of which a smaller value indicates greater activity [27]. Consequently, the IC_50_ values of free phenolic extracts prepared from an aqueous extract of stems and leaves were higher than 3 mg/mL, indicating a lower antioxidant activity. Contrarily, the corresponding IC_50_ values of the ethyl acetate extracts were in lower antioxidant levels, ranging from 0.070 mg/mL in the root samples to 0.699 mg/mL in the leaf samples. The ethyl acetate root extract only required 0.07 mg/mL to reduce the DPPH radicals by 50%. On the other hand, the IC_50_ of the chloroform and hexane extracts in each plant part varied from 0.175 to 1.737 mg/mL.

### 3.3. Antioxidant Activity with the ß-Carotene Bleaching Method

**Figure 2:**
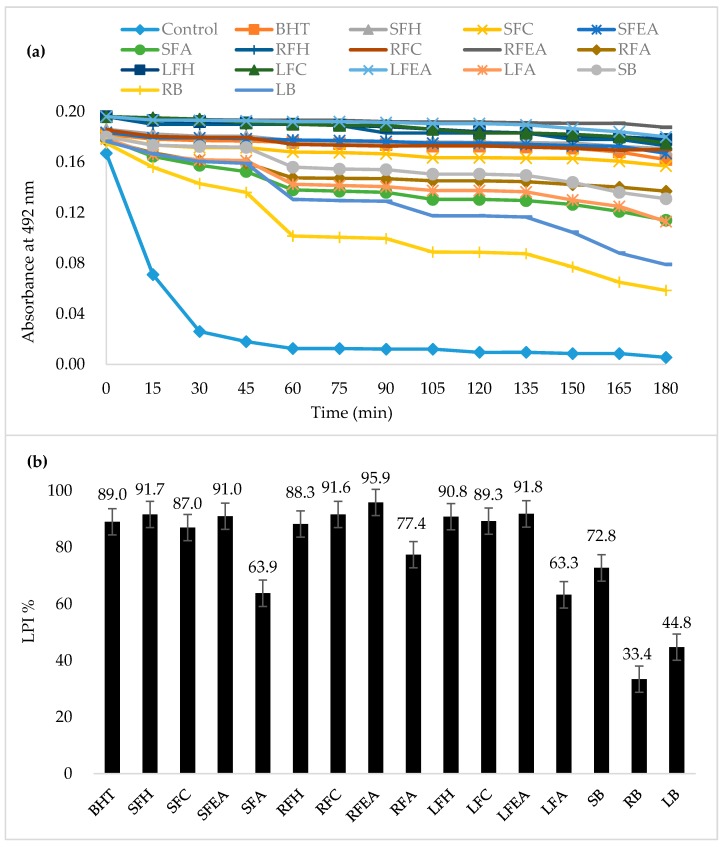
Antioxidant activity of *Phal.* Sogo Yukidian “V3” orchid sample extracts measured by *β*-carotene bleaching method (**a**) and their lipid peroxidation inhibition (%LPI) (**b**) Control (MeOH); BHT: Butylated hydroxytoluene. SFH: Stem free hexane, SFC: Stem free chloroform, SFEA: Stem free ethyl acetate, SFA: Stem free aqueous, RFH: Root free hexane, RFC: Root free chloroform, RFEA: Root free ethyl acetate, RFA: Root free aqueous, LFH: Leaf free hexane, LFC: Leaf free chloroform, LFEA: Leaf free ethyl acetate, LFA: Leaf free aqueous, SB: Stem bound, RB: Root bound, LB: Leaf bound.

Unsaturated *β*-carotene can be damaged by linoleic acid to produce linoleate and other free radicals in a chemical process, namely, *β*-carotene-bleaching; antioxidants are capable of neutralizing these free radicals [16]. Antioxidant tests that used the discoloration of *β*-carotene are widely used, as *β*-carotene is extremely sensitive to intermediate oxidative free radicals [28]. All extracts of *Phal.* Sogo Yukidian “V3” cultivar sampled inhibited *β*-carotene oxidation (Figure 2a). The polar extracts, ethyl acetate, and aqueous solution exhibited superior inhibition to the non-polar extracts, hexane and chloroform, and bound phenolic extracts. The LPI values of sample extracts ranged from 33.4% to 94.2% (Figure 2b). The free linoleic acid is formed when the abstraction of a hydrogen atom from one of the dialytic methyl groups attacks the molecules of unsaturated *β*-carotene. When the molecules of *β*-carotene lose their oxidative double bond, the resulting compound will lose its orange color. The presence of antioxidant activity in various extracts can interfere with the bleaching of *β*-carotene levels by neutralizing the free radicals formed in the system [29].

### 3.4. Reducing Power

**Figure 3:**
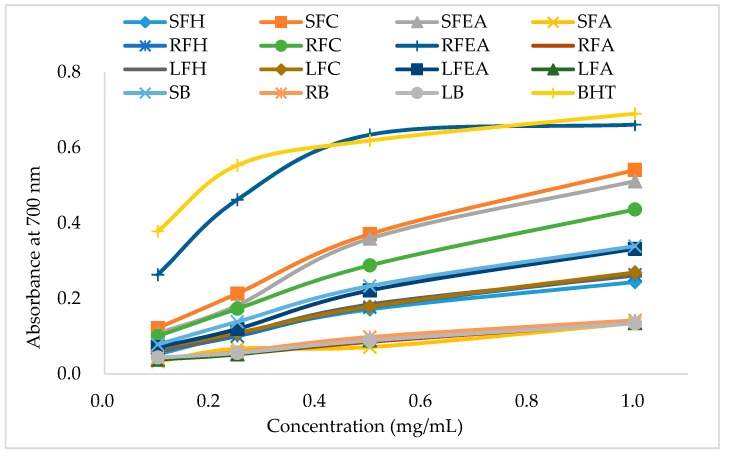
Reducing power of *Phal.* Sogo Yukidian “V3” orchid sample extracts. BHT: Butylated hydroxytoluene. SFH: Stem free hexane, SFC: Stem free chloroform, SFEA: Stem free ethyl acetate, SFA: Stem free aqueous, RFH: Root free hexane, RFC: Root free chloroform, RFEA: Root free ethyl acetate, RFA: Root free aqueous, LFH: Leaf free hexane, LFC: Leaf free chloroform, LFEA: Leaf free ethyl acetate, LFA: Leaf free aqueous, SB: Stem bound, RB: Root bound, LB: Leaf bound.

The potential antioxidant activity of a compound is indicated by its ability to transfer electrons (reducing power). Sample extracts with high antioxidant activity also have a high reducing power [10]. Figure 3 shows the reducing power of hexane, chloroform, ethyl acetate, aqueous, and bound extracts prepared from the stems, roots, and leaves of *Phal*. Sogo Yukidian “V3”. In this assay, the yellow color of the test solution varied between green and blue, depending on the reduction capacity of each compound. The presence of substances arising from the degradation of antioxidants contributed to the reduction of Fe^3+^ to Fe^2+^ [30].

The reducing power of all sample extracts was proportionate to the concentrations applied. The ethyl acetate extracts exhibited a higher ability to reduce Fe^3+^ to Fe^2+^ than those of the chloroform, hexane, aqueous, and bound extracts. For the stem extracts, the reducing power IC_50_ values ranged from 0.871 to 4.495 mg/mL, the IC_50_ values ranged from 1.544 to 4.553 mg/mL for the leaf extracts, and 0.450 to 4.354 mg/mL for the root extracts (Table 3).

### 3.5. HPLC Quantification

The hexane, chloroform, ethyl acetate, aqueous, and bound extracts prepared from the stems, roots, and leaves of *Phal.* Sogo Yukidian “V3” cultivar contained high amounts of phenolic compounds and showed strong antioxidant activity. These extracts were used to identify and quantify the individual phenolic acids.

Using HPLC, five compounds including caffeic acid, syringic acid, vanillin, ellagic acid, and cinnamic acid were detected in varying quantities (Table 4). Among these phenolics, caffeic and ellagic acids were most prevalent in leaves. Caffeic acid (574.75 μg/g DW), syringic acid (16.98 μg/g DW), and cinnamic acid (15.43 μg/g DW) were found only in the hexane extracts of stems, ethyl acetate extracts of roots, and chloroform extracts of stems. Furthermore, vanillin was detected in the ethyl acetate extracts of roots (11.06 μg/g DW) and in aqueous extracts of leaves (51.27 μg/g DW). The highest concentration of ellagic acid (143.90 μg/g DW) was found in ethyl acetate extracts of stems, followed by chloroform extracts of stems (134.02 μg/g DW). Several sources indicate that phenolic acids such as ferulic, sinapic, *p*-coumaric, and ellagic acids are strong antioxidants [31,32,33]. Ellagic acid was reported to obtain anti-inflammatory, anti-proliferating, anti-angiogenesis, anti-cancer, anti-fungal, anti-cancer, and anti-depressant effects [34].

## 4. Discussion

Plants are known to provide substances and chemicals effective in the treatment and remedy of various diseases [35,36,37,38]. Orchids constitute an order of royalty in the world of ornamental plants, are important for large horticulture, and play an important role in balancing forest ecosystems. Orchids are economically important as they are used not only for decorative purposes, but also for the treatment of certain diseases in traditional medicines [35]. Tubers and pseudobulbs of several orchids like *Orchis latifolia*, *Orchis mascula*, *Cymbidium aloifolium*, *Zeuxine strateumatica*, and some species of *Dendrobium*, *Eulophia,* and *Habenaria*, can treat a host of diseases [39]. The roots of *Vanda tessellate* can be used for manufacturing poison antidotes as well as for treating rheumatic and abdominal pain [40]. *Dendrobium fimbriatum* has been used as a treatment for liver upset and nervous debility, while *Dendrobium teretifolium* has been used for headache and pain relief. Hawkes [41] and Withner et al. [42] reported that *Oberonia*, *Eria*, *Bulbophyllum*, *Eulophia*, *Geodorum*, *Grammatophylum*, and *Hetaeria* could be used as a traditional medicine against various diseases.

There are an impressive number of modern medicines that have been isolated from natural sources [43]. In this study, the method of sequential extraction with different solvents (hexane, chloroform, ethyl acetate, and water) revealed a rich presence of flavonoids and phenols. Although, among 15 individual phenolic acids used as standards, there were only five constituents detected by HPLC from the waste materials of the orchid (Table 4). The existence of other individual flavonoids and phenols in low concentrations, and antioxidants other than flavonoids and phenolics, should be investigated by using analytical instruments with higher sensitivity of detection such as gas chromatography-mass spectrometry (GC-MS). Extracts with phenolic substance-medicated antioxidant activity were shown in conjunction with the development of reducing power [44]. As such, the ethyl acetate extract may contain much higher amounts of electron donors for reducing, and they can react with free radicals to convert them into more stable products, terminating the radical chain reaction.

The findings of this study suggest that waste material from orchids resulting from the production process for commercial flowers may be exploited as a natural source of antioxidants. In 2009, Thailand exported $80 million US worth of orchids, of which the *Phal.* cultivar comprised 23% of that total [45]. In 2012, Taiwan was the foremost exporter of the *Phal.* plant (69%) which, of a total of $164.70 million US, the major cultivar was the *Phal.* Sogo Yukidian “V3” [4]. Rich antioxidants were found in the roots, with well-known phenolic compounds—caffeic acid at 574.75 μg/g DW, and ellagic acid at 328.07 μg/g DW—in the stems. In this study, extract solvents of increasing polarity were used to examine their capacity for extracting antioxidants. It was found that ethyl acetate extracts of the roots provided maximum antioxidant yield. In general, polar solvent extracts showed greater antioxidant yield than others that were studied. One explanation is that highly polar solvents often accumulate free substances with a hydroxyl moiety, such as phenols and flavonoids [46,47,48]. This suggests that the root of the orchid was rich in compounds with the hydroxyl moiety, although only five free individual phenolic acids were detected by HPLC. Further analysis should be concentrated on the roots of the *Phal*. plant to exploit its potent antioxidant capacity.

Results of this study may be worthwhile to the principal locations of orchid production such as Taiwan, Thailand, and the Netherlands, where orchids play an active role in flower production, but waste disposal causes environmental problems and are costly to treat [49,50].

## 5. Conclusions

The results of this study verified that ethyl acetate extracts from *Phalaenopsis* Sogo Yukidian “V3” plant parts have potent antioxidant activity and may be utilized as an efficient and safe antioxidant source. Further trials examining other medicinal and pharmaceutical properties, as well as application of further analytical instruments with higher sensitivity to determine potent constituents in the waste of the *Phalaenopsis* should be conducted.

## Figures and Tables

**Table 1:** Names and abbreviations of plant extracts with different solvents.

Name of Plant and Extraction Solvent	Abbreviation
Sogo Yukidian “V3” stem free hexane	SFH
Sogo Yukidian “V3” stem free chloroform	SFC
Sogo Yukidian “V3” stem free ethyl acetate	SFEA
Sogo Yukidian “V3” stem free aqueous	SFA
Sogo Yukidian “V3” root free hexane	RFH
Sogo Yukidian “V3” root free chloroform	RFC
Sogo Yukidian “V3” root free ethyl acetate	RFEA
Sogo Yukidian “V3” root free aqueous	RFA
Sogo Yukidian “V3” leaf free hexane	LFH
Sogo Yukidian “V3” leaf free chloroform	LFC
Sogo Yukidian “V3” leaf free ethyl acetate	LFEA
Sogo Yukidian “V3” leaf free aqueous	LFA
Sogo Yukidian “V3” stem bound	SB
Sogo Yukidian “V3” root bound	RB
Sogo Yukidian “V3” leaf bound	LB

**Table 2:** DPPH radical scavenging activity of *Phal.* Sogo Yukidian “V3” cultivar extracts in terms of IC_50_ values.

Samples	DPPH IC_50_ (mg/mL)		
	Stems	Roots	Leaves
Hexane	1.634 ± 0.020 ^b^	1.663 ± 0.083 ^a^	ns
Chloroform	0.175 ± 0.002 ^d^	0.210 ± 0.005 ^d^	1.737 ± 0.040 ^b^
Ethyl Acetate	0.195 ± 0.001 ^d^	0.070 ± 0.011 ^e^	0.699 ± 0.009 ^d^
Aqueous	3.197 ± 0.046 ^a^	1.537 ± 0.007 ^b^	6.697 ± 0.025 ^a^
Bound Extract	0.377 ± 0.007 ^c^	0.959 ± 0.054 ^c^	1.348 ± 0.134 ^c^
BHT	0.021 ± 0.012		

Values represent means ± SD (standard deviation). Values with no letter in common in each column are not significantly different (*p* < 0.05) (*n* = 3); Different lowercase letters denote significant differences in the same column; ns: not significant; BHT: Butylated hydroxytoluene.

**Table 3:** Reducing power of *Phal.* Sogo Yukidian “V3” orchid in terms of IC_50_ values.

Samples	IC_50_ (mg/mL)		
	Stems	Roots	Leaves
Hexane	2.191 ± 0.025 ^b^	2.018 ± 0.013 ^b^	2.029 ± 0.037 ^b^
Chloroform	0.871 ± 0.012 ^d^	1.143 ± 0.019 ^c^	2.020 ± 0.022 ^b^
Ethyl acetate	0.929 ± 0.007 ^d^	0.450 ± 0.005 ^d^	1.544 ± 0.067 ^c^
Aqueous	4.495 ± 0.051 ^a^	4.354 ± 0.087 ^a^	4.312 ± 0.112 ^a^
Bound extract	1.523 ± 0.068 ^c^	4.167 ± 0.105 ^a^	4.553 ± 0.099 ^a^
BHT	0.262 ± 0.005		

The values presented represent means ± SD (standard deviation) (*p* < 0.05). Values with no letter in common in each column are not significantly different (*n* = 3); Different lowercase letters denote significant differences in the same column; BHT: Butylated hydroxytoluene.

**Table 4:** Phenolic acids in *Phal.* Sogo Yukidian “V3” cultivar.

Samples	Phenolic Contents (μg/g DW)				
	Caffeic Acid	Syringic Acid	Vanillin	Ellagic Acid	Cinnamic Acid
SFH	574.75 ± 3.87	-	-	50.15 ± 5.30 ^b^	-
SFC	-	-	-	134.02 ± 0.14 ^a^	15.43 ± 0.10
SFEA	-	-	-	143.90 ± 0.14 ^a^	-
SFA	-	-	-	-	-
RFH	-	-	-	-	-
RFC	-	-	-	7.05 ± 1.41 ^d^	-
RFEA	-	16.98 ± 10.35	11.06 ± 1.22	21.09 ± 21.09 ^c^	-
RFA	-	-	-	11.00 ± 0.02 ^cd^	-
LFH	-	-	-	-	-
LFC	-	-	-	71.08 ± 0.09 ^b^	-
LFEA	-	-	-	-	-
LFA	-	-	51.27 ± 0.09	-	-
SB	-	-	-	-	-
RB	-	-	-	-	-
LB	-	-	-	-	-
			ns		

Values presented represent the mean ± SD. Values with no letter in common in each column are not significantly different (*p* < 0.05) (*n* = 3). Different lowercase letters denote significant differences in the same column; ns: not significant. SFH: Stem free hexane, SFC: Stem free chloroform, SFEA: Stem free ethyl acetate, SFA: Stem free aqueous, RFH: Root free hexane, RFC: Root free chloroform, RFEA: Root free ethyl acetate, RFA: Root free aqueous, LFH: Leaf free hexane, LFC: Leaf free chloroform, LFEA: Leaf free ethyl acetate, LFA: Leaf free aqueous, SB: Stem bound, RB: Root bound, LB: Leaf bound.
